# A Case of Venereal Consumption Cured by Mercurial Frictions

**Published:** 1781-02

**Authors:** 


II. *A Case of Venereal Consumption cured by Mercurial Frictions.* *By* S. Theophilus de Meza, M. D. *(From the* Acta Havnienſia, Vol. II.)

ABOUT twenty years ago, C. B. a young gentleman, 25 years of age, contracted the lues venerea. He had ulcers in different parts of his body, particularly in his mouth; and exoſtoſis of the os frontis; to theſe ſymptoms were added hectic fever, colliquative ſweats, diarrhœa, a conſtant cough, an expectoration of pus, eſpecially towards morning, and in a word, all the marks of a confirmed phthiſis pulmonaris.

He had been under the care of a practitioner of great reputation and experience, but the remedies preſcribed having failed of ſucceſs, he thought proper to apply to me. He was at this time in a very emaciated ſtate, but was anxious to undergo a courſe of mercury. I was fearful of giving it to him internally, leſt by ſtimulating the bowels it ſhould increaſe the diarrhœa, and thus haſten his death. I therefore began with rubbing a ſmall quantity of mercurial ointment every night on his arms and legs alternately. In a very few days his mouth began to be affected, but as the ſalivation increaſed, the diarrhœa, and ſweats were perceived to leſſen, and his ſtrength ſeemed rather to increaſe than diminiſh.

I continued the frictions till the patient ſpit about a pound of ſaliva in the ſpace of about four-and-twenty hours, and at the end of eight weeks, I had the ſatisfaction to find him free from every ſymptom of lues and phthiſis. He recovered his health perfectly, and is now the father of ſeveral healthy children.

